# Editorial: Cardiovascular health and cognitive aging

**DOI:** 10.3389/fepid.2023.1253694

**Published:** 2023-11-08

**Authors:** Jingkai Wei, Donglan Zhang

**Affiliations:** ^1^ Department of Epidemiology and Biostatistics, Arnold School of Public Health, University of South Carolina, Columbia, SC, United States; ^2^ Division of Health Services Research, Department of Foundations of Medicine, New York University Long Island School of Medicine, New York, NY, United States; ^3^ Department of Population Health, New York University Grossman School of Medicine, New York, NY, United States

**Keywords:** cardiovascular health, dementia, cognitive aging, risk factors, epidemiology

**Editorial on the Research Topic** Cardiovascular health and cognitive aging

Dementia has become a major public health issue in the U.S (1). It is well known that cardiovascular disease shares numerous risk factors with dementia (2–4). An increasing body of evidence suggests that cardiovascular health plays a crucial role in cognitive aging. The five studies we collected in the Research Topic *Cardiovascular Health and Cognitive Aging* provide compelling insights into the topic.

The first study by Molloy et al. examines the associations of the heart rate, heart rate variability, and cognition in cognitively healthy individuals with pathological amyloid/tau ratio in cerebral spinal fluid, as there is a gap in knowledge on the relationship in older individuals with early Alzheimer's disease pathology. The results indicate that cognitively healthy individuals with pathological amyloid/tau ratio with a higher resting heart rate showed lower scores on the Mini-Mental State Examination, a common test for cognitive function, and less brain activation during a Stroop interference task, a cognitive task requiring attention and cognitive control. Also, vagally mediated heart rate variability, a specific type of heart rate variability, was significantly associated with task switching accuracy in cognitively healthy individuals with normal amyloid/tau ratio, but not in cognitively healthy individuals with pathological amyloid/tau ratio. The findings of Xu et al. utilizing Mendelian randomization, further highlight the relationship between cardiovascular health and cognitive aging, revealing that cognitive impairment can be a causal risk factor for coronary artery disease. Together, these studies reinforce the idea that the heart and brain are physiologically interconnected, and the relationship is bidirectional, implying that any strategies for preserving cognitive function should consider cardiovascular health, and vice versa.

The study by Liu et al. delivers a message of the escalating global burden of atrial fibrillation/flutter, particularly in low- to middle-sociodemographic index regions. It warns of the impending surge in atrial fibrillation/flutter-associated death and highlights the critical role of high systolic blood pressure and body mass index as significant contributors. Given the strong association between cardiovascular health and cognitive function, it is reasonable to anticipate that the surge in atrial fibrillation/flutter may subsequently influence cognitive aging, particularly in vulnerable populations.

Sleep is a lifestyle factor that could potentially moderate the link between cardiovascular health and cognitive aging, and has been newly added as an important component to the “Life's Essential 8”, which defines cardiovascular health (5). In the study by Li et al. both short and long sleep durations are associated with higher all-cause and cardiovascular mortality in the National Health and Nutrition Examination Survey. As sleep is also known to play a crucial role in cognitive function (6), this article suggests that optimizing sleep duration might be a viable strategy to not only improve cardiovascular health but also mitigate cognitive decline.

The final study by Brain et al. provides a broad perspective on the associations between cardiovascular diseases and dementia. The associations of heart disease, heart failure, atrial fibrillation, with all-cause dementia, coupled with the complex relationships involving hypertension and cholesterol, emphasize the need for targeted cardiovascular disease and its risk factors for strategies of dementia risk reduction.

In sum, the research articles discussed above underscore the intricate interplay between cardiovascular health and cognitive aging. It calls for a holistic approach that takes demographic factors, lifestyle elements like sleep, and specific cardiovascular disease risk factors into account to tackle the global issue of cognitive aging. Given the complexity involved, there is an urgent need for collaborative efforts among clinicians and public health professionals to optimize cardiovascular health as a means of promoting healthy cognitive aging. Future research should focus on unraveling the underlying mechanisms that link heart and brain health, as well as to identify the most effective strategies for dementia risk reduction and better cognitive health.
